# Supplementary figures and images for: Proteomic identification of the UDP-GlcNAc: PI α1–6 GlcNAc-transferase subunits of the glycosylphosphatidylinositol biosynthetic pathway of Trypanosoma brucei
Source: PLoS One. 2021 Mar 18;16(3):e0244699. doi: 10.1371/journal.pone.0244699 (PMC7971885; doi:10.1371/journal.pone.0244699)

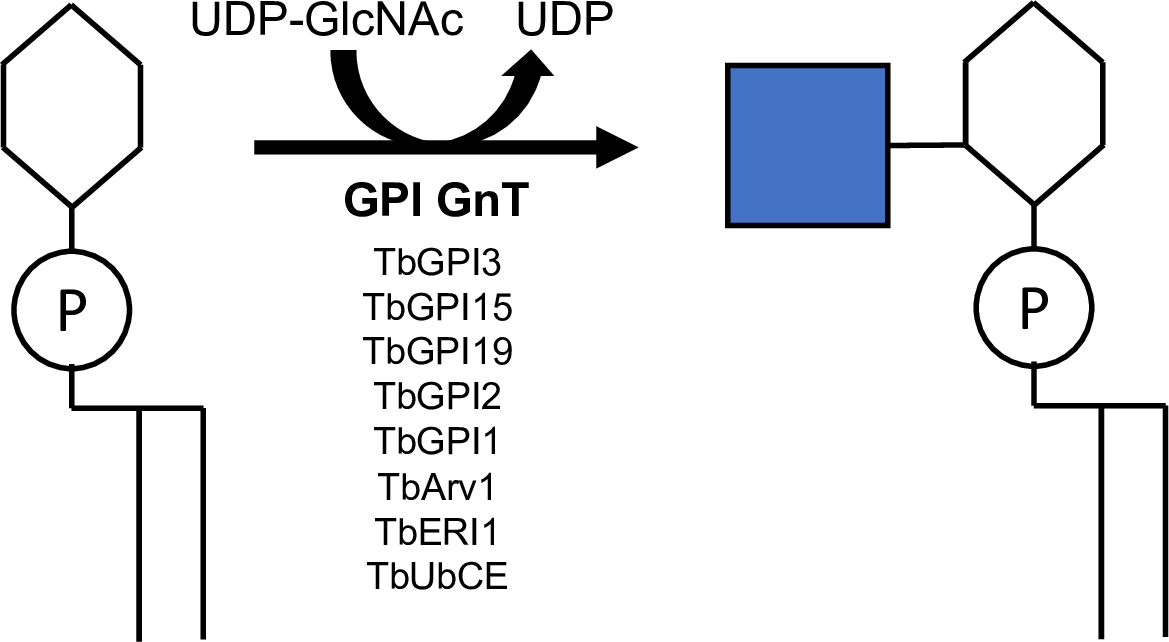

Supplement: S1 Graphical abstract — (TIF) [file pone.0244699.s002.tif]
