# Supplementary material for: Proteomic identification of the UDP-GlcNAc: PI α1–6 GlcNAc-transferase subunits of the glycosylphosphatidylinositol biosynthetic pathway of Trypanosoma brucei
Source: PLoS One. 2021 Mar 18;16(3):e0244699. doi: 10.1371/journal.pone.0244699 (PMC7971885; doi:10.1371/journal.pone.0244699)
Supplement: S1 Raw images — (PDF) [file pone.0244699.s005.pdf]

a. Fig 1 B, C

1 2 3 4 5 1 2 3 4 5

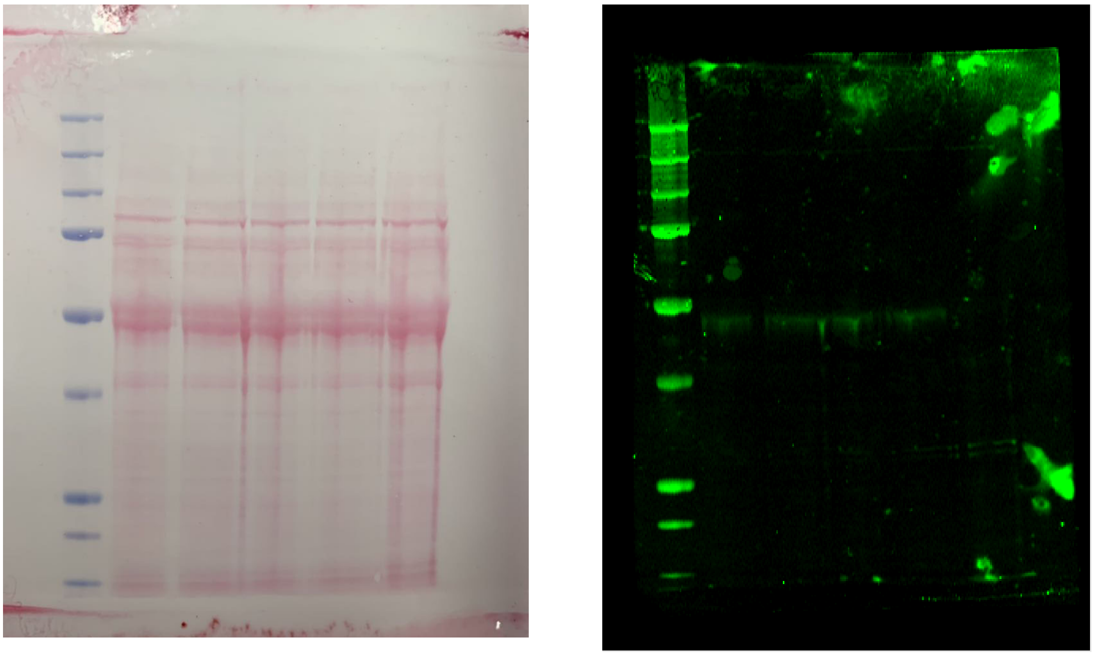

Ponceau staining Western blotting

1. TbGPI3-3Myc clone 1
2. TbGPI3-3Myc clone 2
3. TbGPI3-3Myc clone 3
4. TbGPI3-3Myc clone 4
5. Wild type control

b. Fig 2 A, B

1 2 3 4 5 6 1 2 3 4 5 6

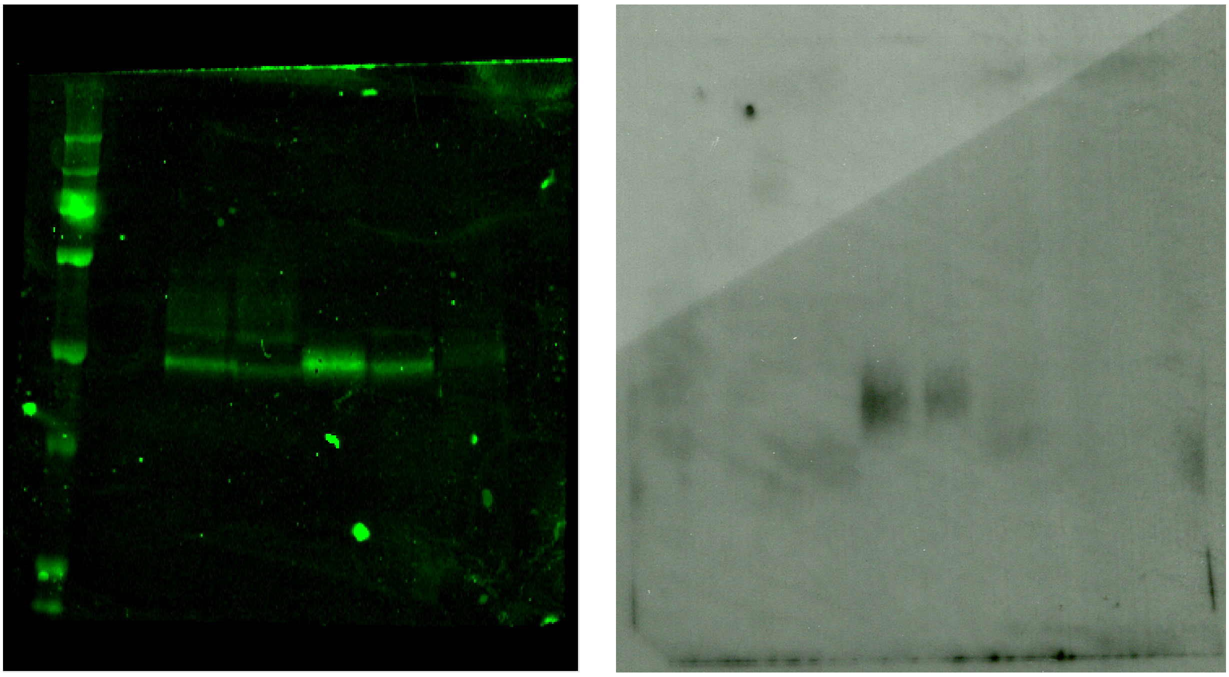

1. Wild type cells lysed with 1% TX-100 following IP with Myc-Trap beads
2. TbGPI3-3Myc cells lysed with 0.5% digitonin following IP with Myc-Trap beads
3. TbGPI3-3Myc cells lysed with 01% digitonin following IP with Myc-Trap beads
4. TbGPI3-3Myc cells lysed with 1% TX-100 following IP with Myc-Trap beads
5. TbGPI3-3Myc cells lysed with 1% NOG following IP with Myc-Trap beads
6. TbGPI3-3Myc cells lysed with 1% DM following IP with Myc-Trap beads
