# Supplementary material for: Proteomic identification of the UDP-GlcNAc: PI α1–6 GlcNAc-transferase subunits of the glycosylphosphatidylinositol biosynthetic pathway of Trypanosoma brucei
Source: PLoS One. 2021 Mar 18;16(3):e0244699. doi: 10.1371/journal.pone.0244699 (PMC7971885; doi:10.1371/journal.pone.0244699)
Supplement: S1 Fig — (PDF) [file pone.0244699.s001.pdf]

```
GPI3            MG------------------------------FNIAMLCDFFYPQLGGVE
PIGA            MACRGGAGNGHRASATLSRVSPGSLYTCRTRTHNICMVSDFFYPNMGGVE
TbGPI3          MR-----------------------------RHRVAVVSDFFYPGFGGVE
                *                               ..:.::.***** :****

GPI3            FHIYHLSQKLIDLGHSVVIITHAYKDRVGVRHLTNGLKVYHVPFFVIF--
PIGA            SHIYQLSQCLIERGHKVIIVTHAYGNRKGIRYLTSGLKVYYLPLKVMY--
TbGPI3          VHIYSLGQCLMRRGHKVIVITRAYGDTCGVRYLTNGMKVYYLPLMAVKLP
                 *** *.* *:  **.*:::*:** :  *:*:**.*:***::*: .:

GPI3            -RETTFPTVFSTFPIIRNILLREQIQIVHSHGSASTFAHEGILHANTMGL
PIGA            -NQSTATTLFHSLPLLRYIFVRERVTIIHSHSSFSAMAHDALFHAKTMGL
TbGPI3          AGSVTLPTMYLTFATMRSIFIRERITVVHGHQNTSNLCHEALFHAGTLGL
                  . * .*:: ::. :* *::**:: ::*.* . * :.*:.::** *:**

GPI3            RTVFTDHSLYGFNNLTSIWVNKLLTFTLTNIDRVICVSNTCKENMIVRTE
PIGA            QTVFTDHSLFGFADVSSVLTNKLLTVSLCDTNHIICVSYTSKENTVLRAA
TbGPI3          KTCFTDHSLFGFADVSSIHINKVCEWSLRNVDQVICVSNTSRENTVLRAK
                :* ******:** :::*:  **:   :* : :::**** *.:** ::*:

GPI3            LSPDIISVIPNAVVSEDFKPRDPTGG--TKRKQSRDKIVIVVIGRLFPNK
PIGA            LNPEIVSVIPNAVDPTDFTPDPF---------RRHDSITIVVVSRLVYRK
TbGPI3          IDPQRVSVIPNATDCSFFTPPDDMKYKSWASKVENEGLTIVVIGRLVYRK
                :.*: :******.    *.*              .: :.***:.**. .*

GPI3            GSDLLTRIIPKVCSSHEDVEFIVAGDGPKFIDFQQMIESHRLQKRVQLLG
PIGA            GIDLLSGIIPELCQKYPDLNFIIGGEGPKRIILEEVRERYQLHDRVRLLG
TbGPI3          GSDLFVDVIPEICKRHPNIRWIVGGDGPRRSQFQQMIERHDLMDRVKMLG
                * **:  :**::*. : ::.:*:.*:**:   :::: * : * .**::**

GPI3            SVPHEKVRDVLCQGDIYLHASLTEAFGTILVEAASCNLLIVTTQVGGIPE
PIGA            ALEHKDVRNVLVQGHIFLNTSLTEAFCMAIVEAASCGLQVVSTRVGGIPE
TbGPI3          SLPHSGVRNVLIQGQIFLNCSLTEAFCIALIEAASCGLLCVSTRVGGVPE
                :: *. **:** **.*:*: ******   ::*****.*  *:*:***:**

GPI3            VLPNEMTVYAEQTSVSDLVQATNKAINIIRSK-ALDTSSFHDSVSKMYDW
PIGA            VLPENLIILCEP-SVKSLCEGLEKAIFQLKSGTLPAPENIHNIVKTFYTW
TbGPI3          VLPPNMLLLAEP-DPSSIITTLEEAIASVP---YISPWELHDNVRRFYRW
                *** :: : .*  . ..:    ::**  :       . .:*: *  :* *

GPI3            MDVAKRTVEIYTNISSTSSADDKDWMKMVANLYKRDGIWAKHLYLLCGIV
PIGA            RNVAERTEKVYDRVSVEAVLPMDK---RLDRLISHCGPVTGYIFALLAVF
TbGPI3          DWVAERTERVYDKIMCTKSPSLYE---RLMNYAS-VGCVYGVICWLLCIG
                  **:** .:* .:         .    : .  .  *     :  *  :

GPI3            EYMLFFLLEWLYPRDEIDLAPKW--PKKTVSNETKEARE----------T
PIGA            NFLFLIFLRWMTPDSIIDVAIDATGPRGAWTNNYSHSKRGGENNEISETR
TbGPI3          DWLMLTFLEFWFPSELIDIAPDF--PLELYSRNREKLQVVGSPS------
                ::::: :*.:  * . **:* .   *    :.: .. :
```

S1 Fig. Sequence alignment of GPI3, PIGA and TbGPI3. The alignment was generated by the Tcoffee program, * = identical residues, : = conserved residues. GPI3, PIGA and TbGPI3 sequences are from Uniprot; GPI3 (P32363 GPI3_YEAST) PIGA (P37287 (PIGA_HUMAN)); TbGPI3 (Q585S5 (Q585S5_TRYB2)).
