# Supplementary material for: Proteomic identification of the UDP-GlcNAc: PI α1–6 GlcNAc-transferase subunits of the glycosylphosphatidylinositol biosynthetic pathway of Trypanosoma brucei
Source: PLoS One. 2021 Mar 18;16(3):e0244699. doi: 10.1371/journal.pone.0244699 (PMC7971885; doi:10.1371/journal.pone.0244699)
Supplement: Attachment — Submitted filename: Response to reviewers_170221.docx [file pone.0244699.s006.docx]

We thank the reviewers for their helpful comments. **We respond in bold** *to their specific points (in italics below)*.

**Reviewer 2 appears satisfied with the paper concluding that** *“[the paper] will provide a basis for further studies to better understand regulation of GPI biosynthesis in not only in T. brucei but also in human and yeast cells. The manuscript is clearly written and the conclusions are sufficiently supported by experimental evidence.”*

**Similarly, Reviewer 4 concludes that** *“The analyses are expertly performed and the finding that the T. brucei complex contains Arv-1 and UbCE homologues has implications for understanding the role of this complex in regulating GPI biosynthesis in crown eukaryotes. Overall, this is a useful study that will be of interest to the parasitology and glycobiology fields*.”

**Other than typographical changes, (such as correcting TbGPI9 to TbGPI19 throughout) no responses appear to be necessary to the reviews of Reviewer 2 and Reviewer 4.**

**Reviewer #1 says that:** *The manuscript is a bit thin and borders on the trivial.* **With all due respect, we would describe the paper as concise and commensurate rather than thin. In responding to the reviewer’s helpful comments (below), we now emphasise just how low-abundance the GPI GnT complex is (see new version of Table 1) and modified text on lines 246-252 & 355-358. We hope this illustrates the non-trivial nature of the optimisations and analyses required, and of the original observations made (*viz* identifying 6 presumed but unproven complex components and 2 new components in a single study). Anecdotally, two groups (one working on trypanosomes and another on mammalian GPI biosynthesis) have already been in touch to comment on how our discovery of Arv-1 as a GPI GnT component has significantly influenced their research directions.**

*Surely the authors could have validated and extended their data to make the paper more generally useful to the field? Some suggestions:*
*(i) Arv1 is new here - an Arv1 knockdown strain could be generated to test whether the complex as seen on native page is affected in any way. This could also be done with one or other of the subunits - perhaps such strains are available and it would be a matter of expressing myc-tagged Gpi3 in these as a reporter.* **Unfortunately, there are no mutant strains available for any of the GPI GnT subunits in which to express TbGPI3-Myc to perform these experiments. The lack of orthogonal confirmation that TbArv1 is physically associated with TbGPI3 is acknowledged as a limitation of the study in the Discussion (lines 353-358).**

*(ii) Is the complex stoichiometric - one copy of each subunit? The native page result (Fig 2) is suggestive, but not definitive. This could be investigated.* **With respect, determining sub-unit stoichiometry in a complex of such low abundance is beyond the scope of this study. We make no claims about stoichiometry in the paper for this reason. This is acknowledged as a limitation of the study in the Discussion (lines 353-358).**

*(iii) PIG-A is a single-pass, tail anchored protein, but TbGpi3 appears to have two transmembrane sequences - this could be checked.* **The reviewer is correct that, according to predictors like TMHMM, TbGPI3 has a putative transmembrane domain close to its N-terminus as well as, like PIG-A, one close to its C-terminus. However, with respect, the purpose of the work in the paper was not to study the topologies of GPI GnT subunits but, as the title suggests, only to identify subunits of the complex.** **This is acknowledged as a limitation of the study in the Discussion (lines 353-358).**

Minor comments:
*line 78 - cite Sobering et al. (2004) Cell for additional subunit Eri1.* **We thank the reviewer for pointing out this omission, the reference is now included (new ref [24]).**

*Figure 1B, C - the Ponceau-stained membrane shows remarkably few bands for a total lysate, with the dominant band running exactly the same as the myc-tagged protein - this seems odd. Th blot in panel C is of quite poor quality - there are clearer results in Fig 2A. Figure 1 seems unnecessary as the key result in this figure is replicated in Fig 2A.* **The Ponceau stain (Fig. 1B) illustrates that we cannot usefully load more material than total lysate of 5E6 trypanosomes because distortions are already beginning to show around 50 kDa. This is because SDS-PAGE patterns of total lysates of bloodstream form *T. brucei* are dominated by disproportionately abundant (glyco)proteins; specifically the highly abundant VSG [1E7 copes per cell, 10% of total cellular protein] that runs just above the 50 kDa marker and tubulin [from the parasite’s densely packed subpellicular microtubule cytoskeleton] that runs just below the 50 kDa marker. Other proteins that have similar apparent molecular weights to these dominant proteins are similarly distorted by them (like TbGPI3-Myc). The absence of any fluorescent signal in Fig. 1C lane 5 (the un-transfected parent cell line) provides evidence of the specificity of the signal. The ‘poorness’ of the Western blot of whole lysate is simply a function of the extremely low abundance of the *in-situ* tagged (not overexpressed) TbGPI3-Myc product, which is indeed why it was necessary to do a pull-down (from 2E8 cells) to have sufficient signal to work with in the experiments in Fig. 2. Please also see our reply to the next of the reviewer’s points. We have taken the reviewers comments on board and now explain the data better in the revised manuscript (see lines 245-252). We respectfully request we retain the data in (Fig 1) now they are better explained.**

*line 251 - it is not necessary to do a pull-down in order to detect complexes in native page - a total lysate could have been run, but perhaps the sample was too dilute?* **The reviewer is correct, the GPI GnT complex is *very* low abundance requiring the pull-down/detection approach. An extremely deep total parasite proteome that identified 7148 protein groups – derived from the data in ref [33] and described in the Materials and Methods under ‘Data Analysis’ – allows us to rank each protein from most (rank 1) to least (rank 7148) abundant. Any undetected proteins are ranked 7149. When these total proteome rankings are listed next to the top-8 proteins detected in the TbGPI3-3Myc pull-down (see new version of Table 1) it is, we hope, easier to appreciate how profound the enrichment of all 8 proteins into the pull-downs is. For example, TbArv1 is 7149 (undetectable) in the total proteome but 6^th^ in the (triplicate) TbGPI3-3Myc pull downs. This is now discussed in lines 353-358.**

*paragraph beginning on line 334 - Arv1 is not essential in yeast cells grown at 25C (Swain, Stukey et al. (2002) JBC; Georgiev, Johansen et al. (2013) Traffic). Although Arv1 may play a role in GPI biosynthesis, perhaps a regulatory role, it is clearly not necessary as otherwise the cells would be inviable. This point should be made clear.* **These references [39,40] are now included and the reviewer’s helpful point is now made clear in the revised text (lines 360-361).**

**Reviewer 3 concludes that** *“The authors identified Arv1 and UbCE proteins as part of GPI-GnT, in addition to TbGPI15, TbGPI9, TbGPI2, TbGPI1, and TbERI1. It is a new finding that two additional factors are componets of GPI-GnT in T. brucei.”* **but comments** *“However, the reviewer asks the authors to confirm the mass spectrometric result before publication”* **because** *“The major concern is whether Arv1 and UbCE are bound with GPI-GnT directly. The authors only detected two proteins with mass spectrometry. The reviewer suggests to validate the mass spectrometric results. It is possible to detect the interactions between tagged Arv1 or UbCE and TbGPI3-3Myc using western blotting.”* **We respectfully suggest that dual-tagging and Western blot confirmation of co-precipitation is not necessary. TbArv1 has been enriched in the TbGPI3-Myc3 pull-downs from being undetectable by proteomics (>7148^th^ in the total proteome) to being the 6^th^ most abundant protein in the TbGPI3-Myc3 triplicate pull downs (see also response to Reviewer 1, above). This is now emphasised in the modified version of Table 1 and in the text on lines 356-358. The lack of orthogonal confirmation of TbArv1 – TbGPI3 association is acknowledged as a limitation of the study (lines 353-358).**

*In lines 340 to 342, the authors wrote that “The complementation of yeast Arv1 mutants by the human Arv1 [38] and recent findings that human Arv1 mutations lead to deficiencies in GPI anchoring [39] [40] strongly suggest a related role in mammalian cells and that it is a component of the mammalian UDP-GlcNAc : PI 1-6 GlcNAc-transferase complex.” It is obvious that Arv1 is involved in the GPI biosynthetic pathway, but cannot specify to be the component of GPI-GnT from the reports. The authors should rewrite the part.* **We have rewritten this section in-line with the reviewer’s helpful comments – see lines 367-369.**

*In yeast, it is reported that Arv1 is required for flipping of GPI intermediates or for efficient synthesis of Man1GlcN-acylPI (Okai et al. (2020) FEBS Lett. 594: 2431; Kajiwara et al. (2008) Mol. Biol. Cell 19: 2069). The difference in GPI biosynthetic pathway between yeast and T. brucei is the flipping steps of GPI intermediates. Only the GlcN-PI across the ER lipid bilayer in yeast GPI biosynthesis, whereas several GPI intermediates seem to be flipped/flopped in T. brucei. The authors need to describe the difference of the flipping reaction in yeast and T. brucei GPI biosynthesis, in addition to the difference of inositol-acylation and mannosylation steps*. **The differences in flipping are now described and referenced [47] (lines 373-376).**

*Based on the previous results and current authors data, is it possible that Arv1 may function as a scaffold for the initial GPI biosynthetic enzymes including GPI-GnT, GPI-deacetylase, and flippase? The authors could discuss such possibilities*. **These possibilities are now discussed (lines 376-378).**
